# Risk factors for incarceration in groin hernia: a prospective observational study

**DOI:** 10.1007/s10029-025-03331-w

**Published:** 2025-04-12

**Authors:** Hande Kandemir, Turgut Donmez, Ahmet Surek, Alpen Yahya Gumusoglu, Mehmet Karabulut, Ozden Canoz, Arif Kaya

**Affiliations:** 1 Department of General Surgery, Adiyaman Kahta State Hospital, Adiyaman, Turkey; 2 Department of General Surgery, Bakirköy Dr. Sadi Konuk Research and Training Hospital, Istanbul, Turkey; 3 Department of General Surgery, Medicana Ataköy Hospital, Istanbul, Turkey; 4 Department of General Surgery, Adiyaman Golbasi State Hospital, Adiyaman, Turkey; 5 Department of General Surgery, Adiyaman Kahta State Hospital, Yavuz Selim, Hastane Cd, No:39, Kâhta/Adıyaman, 02400 Turkey

**Keywords:** Groin, Hernia, Incarceration, Inguinal

## Abstract

**Purpose:**

Groin hernia is one of the most common benign pathologies requiring surgical intervention. Incarcerated groin hernia is a cause of serious morbidity and mortality. In our study, we aimed to identify the risk factors for incarceration in patients with groin hernia and to better understand the factors associated with incarceration, we studied patients who applied to our clinic for groin hernia and underwent surgery.

**Methods:**

A prospective observational study was performed, including all patients who applied to the general surgery out patient clinic or emergency department at Bakirköy Dr Sadi Konuk Training & Research Hospital and were diagnosed with groin or incarcerated groin hernia and underwent hernia repair surgery. Patients were divided into two groups; elective surgery and emergency surgery (incarcerated hernia). Multivariate logistic regression was performed to identify risk factors for incarceration.

**Results:**

The study was performed with a total of 654 cases between January 2021 and February 2023. Of these, 79.4% (*n* = 519) had elective surgery and 20.6% (*n* = 135) had emergency surgery. Increase in defect width, which was determined according to the EHS classification, was significantly associated with an incarcerated hernia (ODDS ratio 4.463 and 17.636, respectively). Additionally, female gender, femoral hernia type, chronic cough, and chronic constipation were found to be independent risk factors for incarceration.

**Conclusion:**

Female gender, chronic cough, chronic constipation, femoral hernia type, and increased defect diameter are independent risk factors for urgent surgery and therefore incarceration in groin.

**Trial registration number:**

The ClinicalTrials.gov ID number of the study is NCT04785430.

**Supplementary Information:**

The online version contains supplementary material available at 10.1007/s10029-025-03331-w.

## Introduction

Groin hernia is one of the most common benign pathologies requiring surgical intervention and constitutes a significant portion of outpatient clinic visits [1]. An incarcerated or strangulated hernia describes situations where the hernia sack contents cannot be pushed back into the abdomen or when the blood flow is impaired to these contents [2]. Prolonged incarceration and strangulation are causes of serious morbidity and mortality and require urgent surgical intervention. In this high-volume patient population, identifying factors that increase the risk of hernia incarceration would play a crucial role in patient management and decision-making, planning the priority of elective surgery, particularly in societies which have a relatively long waiting list for surgery.

In this study, we aimed to identify the risk factors for incarceration in patients with groin hernia. To better understand the factors associated with incarceration, we studied patients who applied to emergency department or outpatient clinic for groin hernia and underwent emergency or elective surgery.

## Materials and methods

Following the ethical committee approval, a prospective observational study was conducted over a period of 2 years at the Bakirköy Dr. Sadi Konuk Training and Research Hospital General SurgeryClinic. The study included adult patients diagnosed with groin hernia who presented to the outpatient clinic and those who were diagnosed with incarcerated groin hernia upon emergency department admission and subsequently underwent hernia repair surgery.

Patients were divided into two groups: elective surgery and emergency surgery (incarcerated hernia). The elective surgery group included patients with reducible groin hernia where abdominal contents (e.g., fat, omentum, or intestine) could be pushed back into the abdominal cavity through the defect in the abdominal wall. In contrast, the emergency surgery group included patients with hernia where abdominal contents could not be reduced back into the abdominal cavity through the defect.

Key characteristics of the study are age, body mass index (BMI), gender, current smoking habits, diabetes mellitus (DM), corticosteroid use, laxative use, history of radiotherapy/chemotherapy, collagen disorder, anticoagulant use, personal history of hernia, family history of hernia, American Society of Anesthesiologists (ASA) score and history of previous surgery (none, gastrointestinal, gynecological or other).

**Fig. 1:**
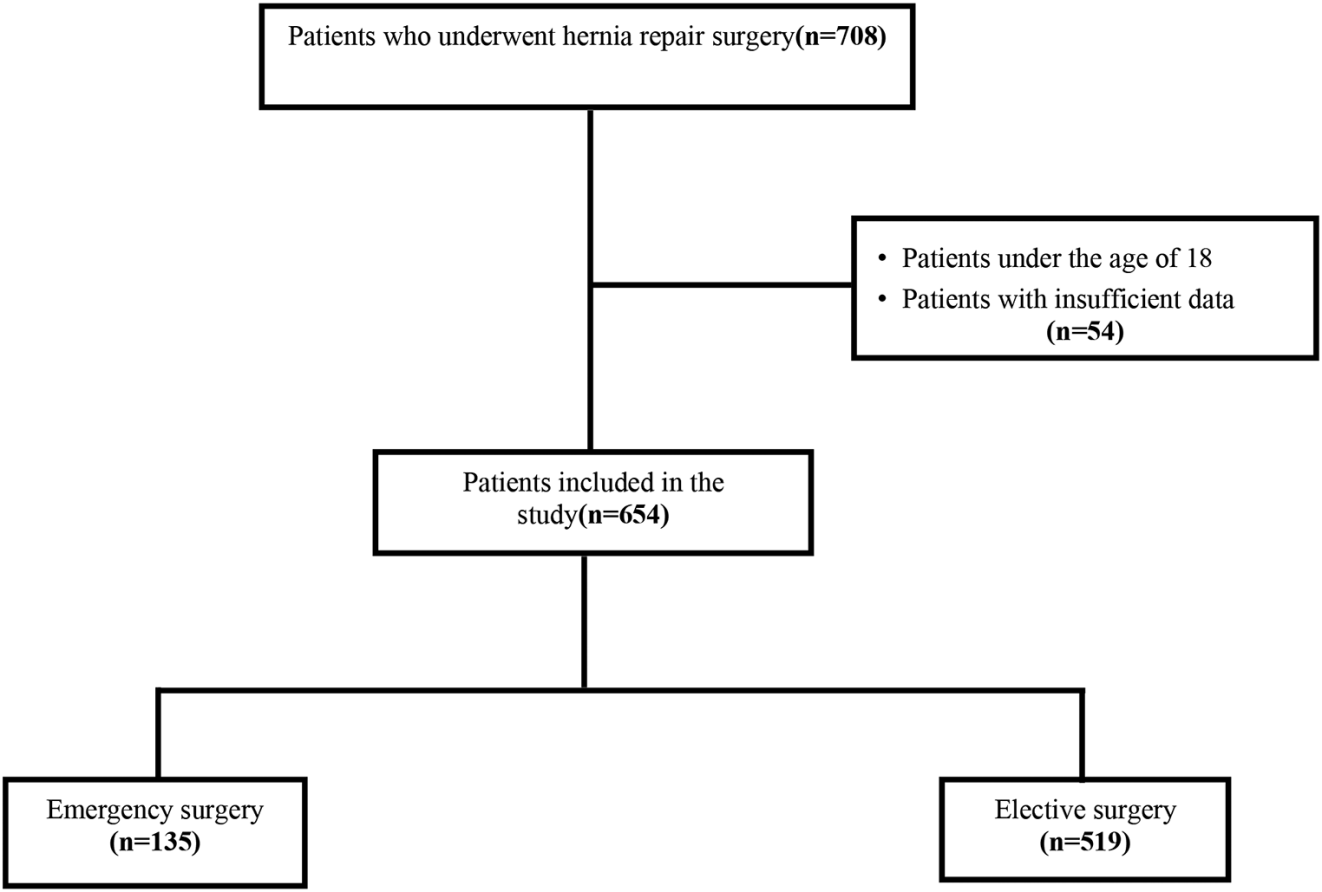
Study design

Factors associated with increased intra-abdominal pressure include; ascites, chronic cough, chronic constipation (defined asfrequent non-defecation episodes lasting more than 3 days), and heavy lifting (defined as lifting more than 10 kg several times a day). Hernia characteristics include; hernia type (direct, indirect, femoral) and defect width, which was measured either through physical examination alone or radiological assessment. Defect width (1.5–2 cm, 2–4 cm, 4–6 cm) was categorized into 3 categories. Patients under 18 years of age, those without complete evaluations and follow-ups, or pregnant patients were excluded from the study (Fig. 1).

This prospective study was conducted with approval from the Health Sciences University Bakırköy Dr Sadi Konuk Health Application and Research Center Ethics Committee in December 2020 with protocol code 2020/539. The ClinicalTrials.gov ID number of the study is NCT04785430.

### Statistical analysis

NCSS (NumberCruncher Statistical System) 2020 Statistical Software (NCSS LLC, Kaysville, Utah, USA) was used for statistical analysis. While evaluating the study data, quantitative variables were shown with mean, standard deviation, median, min and max values, and qualitative variables were shown with descriptive statistical methods such as frequency and percentage. Shapiro Wilks test and Box Plot graphics were used to assess the normality of the data.

Student’s t-test was used for quantitative evaluations of two normally distributed groups.Mann Whitney-U test was used to evaluate variables that did not show normal distribution according to two groups. Chi-Square test, Fisher Exact test and Fisher’s Freeman Halton test were used to compare qualitative data. Logistic Regression Analysis was applied to determine the risk factors affecting the type of surgery. The results were evaluated at the 95% confidence interval and the significance level was *p* < 0.05.

## Results

A total of 708 patients were operated for inguinal hernia at Bakırköy Dr Sadi Konuk Training and Research Hospital between January 2021 and February 2023. 54 of these were excluded from the study due to lack of data (Fig. 1). The study was conducted with a total of 654 cases, 9.5% (*n* = 62) of which were female and 90.5% (*n* = 592) were male. The ages of the cases included in the study ranged between 18 and 93, and the average age was found to be 55.11 ± 15.15. BMI values ​​ranged between 16.1 and 44.9, and the average BMI was determined as 26.51 ± 3.63. 10.4% (*n* = 68) of the cases were ASA I, 69.6% (*n* = 455) were ASA II, 19.9% ​​(*n* = 130) were ASA III and 0.2% (*n* = 1) appears to be ASA IV. When the surgery types are examined; it is seen that 79.4% (*n* = 519) of the patients went through elective surgery and 20.6% (*n* = 135) went through urgent surgery (Table 1).

**Table 1:**
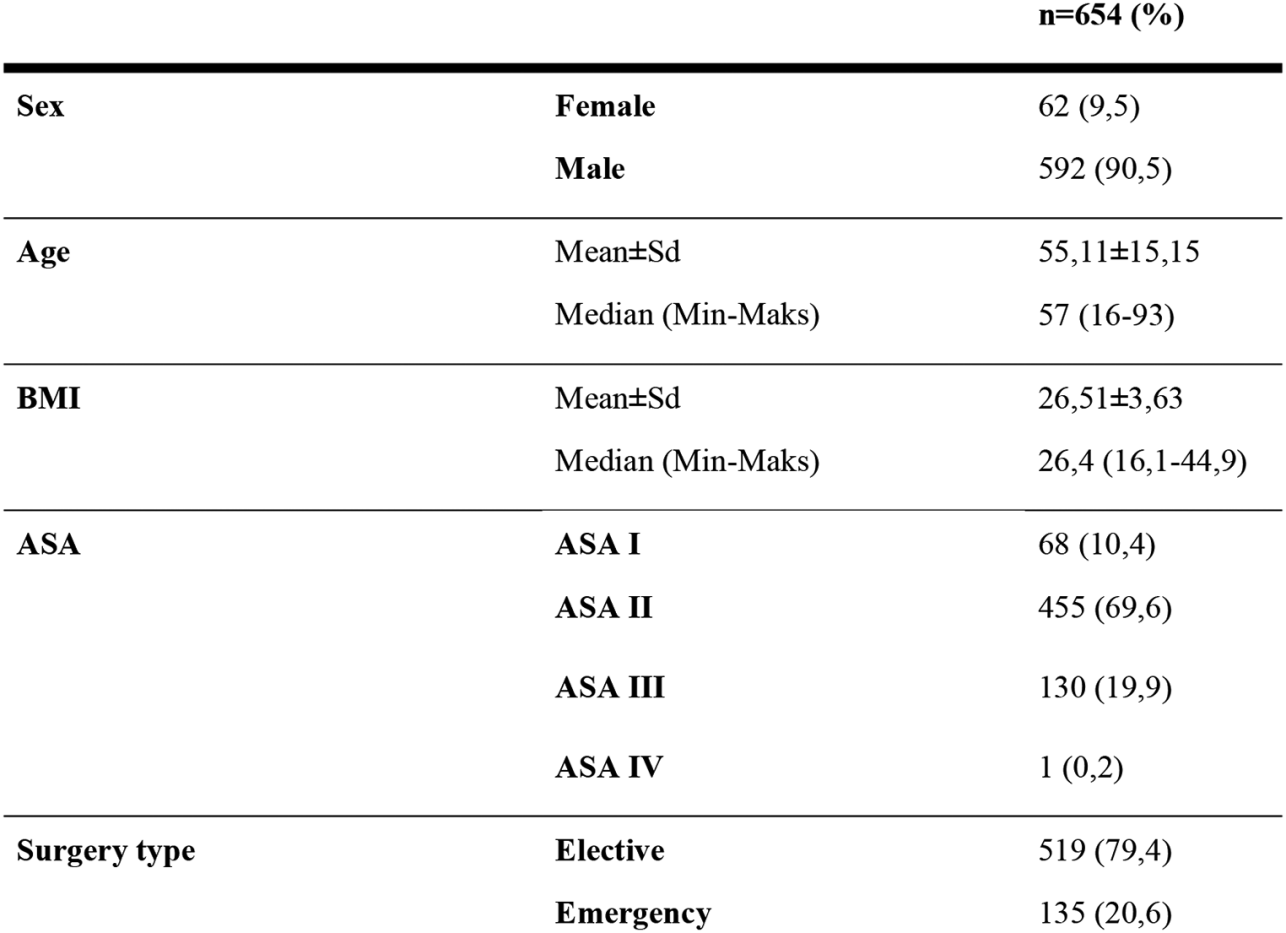
Distributions of patient characteristics

**Fig. 2:**
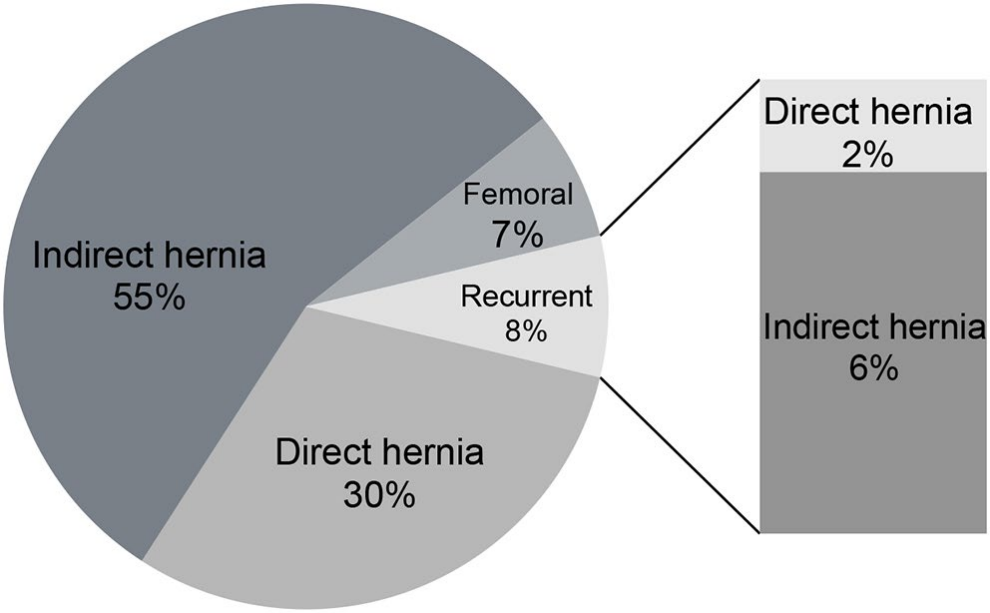
Distribution by Hernia Type

30.2% (*n* = 198) of the patients had direct inguinal hernia, 55% (*n* = 360) had indirect inguinal hernia, and 6.8% (*n* = 45) had femoral hernia. Only7.7% (*n* = 51) of the cases had recurrent hernia (Fig. 2).

The risk of emergency surgery in female cases compared to male cases was found to be significantly higher (ODDS 3.20 (95% CI: 1.85–5.52)).Age and BMI measurements of the cases did not show a statistically significant difference(*p* > 0.05).The number of patients with ASA III score undergoing emergency surgery was found to be statistically significantly higher compared to those undergoing elective surgery (*p* = 0.001; *p* < 0.01).The odds of emergency surgery risk for ASA II compared to ASA I was 1.887 (95% CI: 0.83–4.28); for ASA III, the odds risk was 4.876 (95% CI: 2.06–11.52) (Table 2).

**Table 2:** Comparison of patient characteristics according to operation type

		Surgery type		ODDS Ratio OR (%95 CI)	*p*
		Elective (*n* = 519)	Emergency (*n* = 135)		
Sex	**Female**	36 (6,9)	26 (19,3)	3,20 (1,85 − 5,52)	***0***,001**
	**Male**	483 (93,1)	109 (80,7)		
Age	*Mean ± Sd*	54,67 ± 14,8	56,80 ± 16,38	1,010 (0,99 − 1,02)	***0***,***145***
	*Median (Min-Maks)*	57 (16–92)	59 (22–93)		
BMI	*Mean ± Sd*	26,54 ± 3,43	26,39 ± 4,31	0,989 (0,94 − 1,04)	***0***,***670***
	*Median (Min-Maks)*	26,4 (18,4–41,8)	26,1 (16,1–44,9)		
ASA	**ASA I**	61 (11,8)	7 (5,2)	*Referance*	
	**ASA II**	374 (72,1)	81 (60,0)	1,887(0,83 − 4,28)	***0***,001**
	**ASA III**	84 (16,2)	47(34,8)	4,876 (2,06–11,52)	***0***,001**

*p < 0,05

**p < 0, 01

The risk of emergency surgery in female cases compared to male cases was found to be significantly higher (ODDS 3.20 (95% CI: 1.85–5.52)).Age and BMI measurements of the cases did not show a statistically significant difference(*p* > 0.05).The number of patients with ASA III score undergoing emergency surgery was found to be statistically significantly higher compared to those undergoing elective surgery (*p* = 0.001; *p* < 0.01).The odds of emergency surgery risk for ASA II compared to ASA I was 1.887 (95% CI: 0.83–4.28); for ASA III, the odds risk was 4.876 (95% CI: 2.06–11.52) (Table 2).

The rate of diabetes (DM) in emergency cases was statistically significantly higher than in elective cases (*p* = 0.037; *p* < 0.05), (ODDS 1.696 (95% CI: 1.03–2.80)). In univariate analysis; chronic lung disease was not associated with an incarcerated hernia (*p* > 0.05) but cardiovascular disease was. The odds of emergency surgery risk for those with cardiovascular disease was 1.802 (95% CI: 1.183–2.75)(Table 3).

Cases with a history of abddominal surgery was found to have higher risk of emergency surgery. (*p* = 0.025; *p* < 0.05). The odds for those with a surgical history was 1.546 (95% CI: 1.05–2.67).The percentage of emergency cases with a gastrointestinal (GI) surgery history was also statistically significantly higher (*p* = 0.033; *p* < 0.05), with odds of 1.584 (95% CI: 1.04–2.42). There was no statistically significant differences regarding gynecological and other operations based on surgery type (*p* > 0.05)(Table 3).

**Table 3:** Comparison of patient characteristics according to operation type

		Surgery type		ODDS Ratio OR (%95 CI)	*p*
		Elective(*n* = 519)	Emergency (*n* = 135)		
DM		64 (12,3)	26 (19,3)	*1*,*696 (1*,*03 − 2*,*80)*	***0***,037*
Chronic lung disease		52 (10,0)	14 (10,4)	*1*,*039 (0*,*55 − 1*,*94)*	***0***,***904***
Cardiovascular disease		104 (20,0)	42 (31,1)	*1*,*802 (1*,*183-2*,*75)*	***0***,006**
History of previous surgery		184 (35,5)	62 (45,9)	*1*,*546 (1*,*05 − 2*,*67)*	***0***,025*
	**GI**	107 (21,0)	39 (29,6)	*1*,*584 (1*,*04 − 2*,*42)*	***0***,033*
	**OB/GYN**	9 (1,9)	2 (1,5)	*0*,*765 (0*,*17 − 3*,*53)*	***1***,***000***
	**Other**	68 (13,5)	21 (16,3)	*1*,*249(0*,*74 − 2*,*10)*	***0***,***403***
Covid-19 history		306 (59,0)	53 (39,2)	*0*,*450 (0*,*31 − 0*,*66)*	***0***,001**
Ascites		9 (1,7)	0 (0,0)	*0*,*791 (0*,*76 − 0*,*82)*	***0***,***216***
Chronic cough		46 (8,9)	36 (26,7)	*3*,*793(2*,*29 − 6*,*08)*	***0***,001**
Smoking		161 (31,0)	63 (46,7)	*1*,*946 (1*,*32 − 2*,*86)*	***0***,001**
Laksative use		4 (0,8)	6 (4,4)	*5*,*988 (1*,*66 − 21*,*53)*	***0***,007**
Constipation		21 (4,0)	21 (15,6)	*4*,*368 (2*,*31 − 8*,*27)*	***0***,001**
Anticoagulant use		56 (10,8)	32 (23,7)	*2*,*569 (1*,*58 − 4*,*17)*	***0***,001**
Chemotherapy		3 (0,6)	0 (0,0)	*0*,*793 (0*,*762-0*,*82)*	***1***,***000***
Radiotherapy		2 (0,4)	1 (0,7)	*1*,*929(0*,*17–21*,*44)*	***0***,***501***
Occupation	**Heavy lifting**	125 (24,1)	49 (36,3)	*1*,*79 (1*,*19 − 2*,*69)*	***0***,004**
	**Desk job**	394 (75,9)	86 (63,7)		
Symptom duration (month)	*Mean ± Sd*	15,92 ± 30,48	16,68 ± 22,48	*1*,*00 (0*,*99 − 1*,*01)*	***0***,***105***
	*Median (Min-Maks)*	6 (1-420)	12 (1-130)		
Family history of hernia		67 (12,9)	26 (19,3)	*1*,*61 (0*,*97 − 2*,*65)*	***0***,***060***
History of abdominal wall hernia		94 (18,1)	33 (24,4)	*1*,*46 (0*,*93 − 2*,*29)*	***0***,***098***

*p < 0,05

**p < 0,01

History of Covid-19 was statistically significantly lower in emergency cases (*p* = 0.001; *p* < 0.01). The odds of emergency surgery for those with a Covid history was 0.450 (95% CI: 0.31–0.66). In univariate analysis; chronic cough, smoking, laxative use, constipation, use of anticoagulants were associated with an incarcerated hernia (*p* < 0.01). The rate of heavy work in emergency cases was also significantly higher (*p* = 0.004; *p* < 0.01), with odds of 1.79 (95% CI: 1.19–2.69).There was no statistically significant differences regarding ascites, hernia related symptom duration, history of chemotherapy or radiotherapy (*p* > 0.05)(Table 3).

Looking into hernia characteristics, femoral hernia (PF) and direct hernia (PM) was found to be associated with incarceration. The rate of PF in emergency cases was significantly higher than in elective cases, while the rate of PM was significantly lower (*p* = 0.001; *p* < 0.01). On the other hand, there were no statistically significant differences for indirect or recurrent hernia based on surgery type (*p* > 0.05).

There was a statistically significant relationship between surgery type and physical examination based defect diameters (*p* < 0.01). Patiens with defect diameters of 2 cm and above was significantly more at risk of incarcerated hernia. When taking defect diameters of 1.5–2 cm as a reference, those with defect diameters of 2–4 cm have an emergency surgery risk odds of 3.32 (95% CI: 1.87–5.89), while those with defect diameters of 4–6 cm have arisk of 13.59 (7.43–24.85).There were no significant differences in USG and CT sac diameter and defect diameter based on surgery type (*p* > 0.05) (Table 4).

**Table 4:**
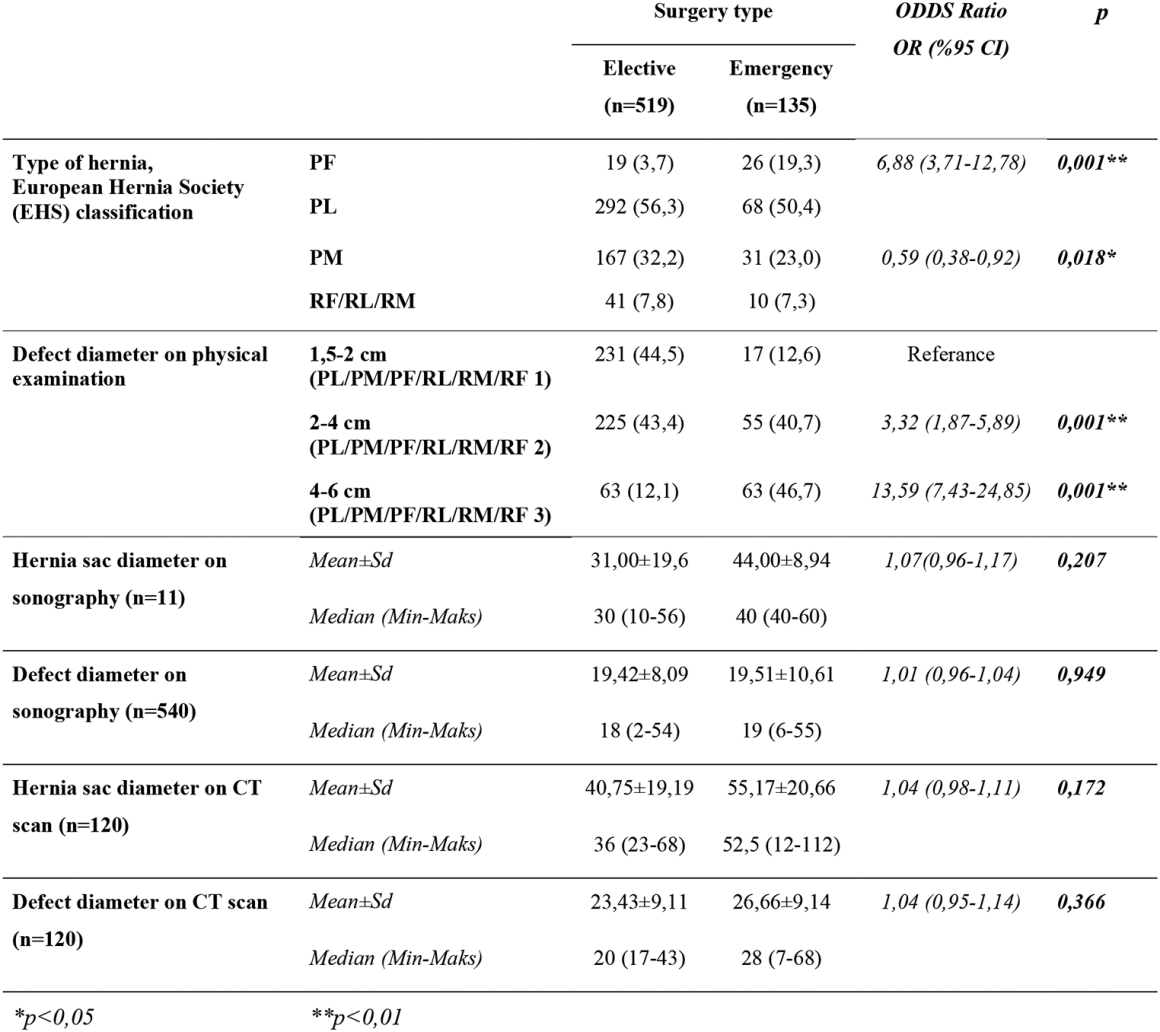
Hernia characteristics

To determine the risk factors affecting surgery type, a multivariate logistic regression analysis was conducted. Significant variables included; gender, ASA, DM, cardiovascular disease, surgical history, Covid history, chronic cough, smoking, laxative use, constipation, anticoagulant use, occupation, direct and femoral hernia types and examination. The model was found to be significant with an explanatory coefficient of 79.4%.

Gender, Covid − 19 history, chronic cough, constipation, femoral hernia type, and defect diameter measurements remained significantly impactful (*p* < 0.05), while other variables were not significant.The odds ratio for the impact of female gender on surgery type was 2.681 (95% CI: 1.24–5.79). The odds ratio for having a history of Covid was 0.511 (95% CI: 0.32–0.81). The odds ratio for having a chronic cough was 2.509 (95% CI: 1.37–4.58). For constipation, the odds ratio was 3.743 (95% CI: 1.58–8.85). The odds ratio for femoral hernia type was 9.612 (95% CI: 3.84–24.03).When defect diameters of 1.5–2 cm are taken as a reference, the odds ratio for defect diameters of 2–4 cm was 4.568 (95% CI: 2.24–9.32); for diameters of 4–6 cm, it was 18.646 (95% CI: 8.83–39.38) (Table 5).

**Table 5:** Logistic regression analysis of risk factors affecting surgery type

	*p*	ODDS	%95 CI	
			Lower	Upper
Sex (female)	***0***,012*	2,681	1,241	5,792
ASA I		Referance		
ASA II	*0*,*740*	1,182	0,441	3,163
ASA III	*0*,*160*	2,201	0,733	6,614
DM	*0*,*275*	0,673	0,330	1,371
Cardiovascular disease	*0*,*449*	0,759	0,372	1,550
History of previous surgery	*0*,*908*	1,042	0,518	2,095
Covid-19 history	***0***,004*	0,511	0,322	0,809
Chronic cough	*0*,003*	2,509	1,372	4,586
Smoking	*0*,*555*	1,194	0,662	2,154
Laksative use	*0*,*508*	1,752	0,333	9,206
Constipation	***0***,003*	3,743	1,583	8,848
Anticoagulant use	*0*,*713*	1,166	0,514	2,646
Occupation (heavy lifting)	*0*,*081*	1,548	0,947	2,532
Direct hernia	*0*,*669*	0,889	0,517	1,527
Femoral hernia	***0***,001*	9,612	3,844	24,034
Defect diameter on physical examination; 1,5–2 cm		Referance		
2–4 cm	***0***,001**	4,602	2,335	9,072
4–6 cm	***0***,001**	18,201	8,897	37,231

## Discussion

Inguinal hernia is one of the most common pathologies requiring surgical intervention in general surgery, affecting %3–8 of the population [1, 2]. Among these, %60 are indirect, and %20–30 are direct inguinal hernias. Femoral hernias occur with a frequency of about %10. The pathophysiology of inguinal hernias is multifactorial [3]. We investigated the impact of demographic characteristics, comorbidities, and hernia characteristics on incarceration. The likelihood of emergency surgery for female patients was found to be significantly higher than for male patients. In a multicenter study by Sneiders et al., which included 4,472 individuals, female sex was associated with incarceration in primary abdominal hernias, although no significant difference was found compared to males. In incisional hernias, the likelihood of emergency surgery for female patients was significantly higher, aligning with our findings [4].

Numerous studies in the literature have examined the relationship between Body Mass Index (BMI) and inguinal hernias. In overweight (BMI > 25–30 kg/m²) and obese (BMI > 30 kg/m²) male patients, a lower incidence of inguinal hernias was observed, suggesting that a higher BMI may have a protective effect [5]. This may be due to the greater amount of abdominal wall fat in overweight patients, providing a stronger and thicker barrier against hernia formation. Another study found that a BMI over 25 kg/m² increased the rate of recurrence in patients undergoing surgery for inguinal hernias [6]. We found no statistically significant relationship between surgery type and BMI measurements.

We did not find a significant relationship between age and emergency surgery. Larger population-based, long-term studies have associated the development of inguinal hernias and incarceration with patient age [5, 7].

In univariate analysis, the number of patients with ASA III risk scores was statistically significantly higher among those who underwent emergency surgery (OR 4.876 [95% CI: 2.06–11.52]). However, in multivariate analysis, when ASA I was taken as the reference, no significant effect was found for ASA II and ASA III scores on incarceration (*p* = 0.160). The literature shows a significant increase in incarceration with higher ASA scores. This may relate to secondary factors not investigated in our study. One possibility is that surgeons may approach elective surgery more cautiously for patients with high ASA scores, leading to these patients presenting to the emergency department with incarcerated inguinal hernias in the long term [4].

Regarding comorbidities, DM and cardiovascular disease was significantly higher in the incarcerated group. However neither variable showed significant difference in multivariate analysis. Chronic lung disease showed no significant difference between groups. In a study by Sneiders et al., the diagnosis of DM significantly increased the rate of incarceration in incisional hernias [4]. In Constance et al.‘s study, the presence of chronic bronchitis and emphysema was investigated for its effects on the development of inguinal hernias, but no statistically significant effect was found [5].

Several studies report increased rates of inguinal hernias following inferior abdominal incisions (especially appendectomy) [8, 9]. In this study, no statistically significant effect of surgical history was found in multivariate analysis.

The presence of a COVID history had an odds ratio of 0.511 (95% CI: 0.32–0.81) regarding the type of surgery (*p* = 0.004). The reason for this relationship remains unclear and requires support from larger volume studies.

One study noted that chronic constipation is associated with an increased risk of incarceration in anterior abdominal hernias, while no significant effects were observed for ascites, chronic cough, history of kidney transplantation, radiotherapy, or smoking [4]. Another found that chronic cough, chronic constipation, and smoking significantly affected the development of inguinal hernias [5]. In our study; chronic cough and chronic constipation were identified as independent risk factors for incarceration. Increased intra-abdominal pressure is a significant factor in hernia pathogenesis, and these variables may increase the risk of incarceration in this context. No significant effect of laxative or anticoagulant use was found on the type of surgery in multivariate analysis. Other patient characteristics; the presence of ascites, history of chemotherapy or radiotherapy showed no statistical significance between groups.

A defect in fibroblast collagen synthesis has been recognized as a factor in the etiology of inguinal hernias [10, 11]. Smoking, which can adversely affect connective tissue metabolism, has been proposed in the literature as a risk factor for inguinal hernia [12] and has been associated with recurrence in one study [13]. In our study, the rate of smoking was significantly higher in incarcerated cases. However, in multivariate analysis, no significant effect was found.

Certain occupations have been identified by various studies as increasing the risk of direct inguinal hernias [1, 14, 15]. Chronic occupational mechanical exposure or prolonged standing may lead to increased intra-abdominal pressure, particularly observed in direct hernias. In a study examining hernia recurrence, 45% of the patients (233 individuals) were reported to work in heavy labor [9]. In another case-control study, total physical activity was not found to be associated with inguinal hernias [16]. In our current study, patients in heavy labor occupations were found to undergo emergency surgery for incarceration significantly more often than those with desk jobs (p: 0.004; *p* < 0.01). In multivariate analysis, no significant effect of occupation type on incarceration was found.

It is known that femoral hernia is associated with higher rates of both recurrence and incarceration, therefore should be treated as soon as possible [5, 17–21]. In our current study, femoral hernia was identified as an independent risk factor for incarceration, consistent with the literature. Duration of the symptoms did not show statistically significant difference.

The ultrasound (US) and computed tomography (CT) measurements for sac diameter and defect diameter did not show statistically significant differences according to the type of surgery (*p* > 0.05). In our clinic, the diagnosis of inguinal hernia is often made through physical examination. Although CT scans are performed in most of the emergency cases, routine US or CT was not employed. Consequently, only a limited number of patients in our sample had defect diameter determined through imaging methods. It is thought that the lack of statistical significance for defect diameter may be attributed to this reason.

In our study, physical examinations were performed according to the European Hernia Society (EHS) classification, and defect diameters were categorized into three groups. When patients classified as PL1, PM1, PF1, RL1, RM1, RF1, with defect diameters of 1.5–2 cm, were taken as the reference value, patients in the PL2, PM2, PF2, RL2, RM2, RF2 group, with defect diameters of 2–4 cm, were found to have a significantly higher risk of emergency surgery. Similarly, patients classified as PL3, PM3, PF3, RL3, RM3, RF3, with defect diameters of 4–6 cm, also demonstrated a significantly higher risk of incarceration compared to the other two groups. In multivariate analysis, an increase in defect diameter measured during physical examination was identified as an independent risk factor for incarceration. Furthermore, the study by Sneiders et al. reported that a defect width of 3–4 cm had the highest odds value for incarcerated hernias (OR 2.85 for primary and OR 2.14 for incisional hernias) [4]. The findings of our study are consistent with the literature, potentially due to the inability of significant abdominal contents to protrude from defects smaller than 2 cm.

## Conclusion

We found that female gender, chronic cough, chronic constipation, femoral hernia type and increased defect diameter are independent risk factors for incarceration and subsequent urgent surgery in groin hernia.

In patients who electively attend a clinic with an inguinal hernia, these factors should be taken into consideration when planning the priority of elective surgery. Studies with a larger patient population are needed to reveal other parameters more clearly.

## Electronic supplementary material

Below is the link to the electronic supplementary material.

Supplementary Material 1
